# Incidence of Gallbladder Stone Formation After Bariatric Surgery Using Ultrasound Imaging in the Southern Region of Saudi Arabia

**DOI:** 10.7759/cureus.25948

**Published:** 2022-06-15

**Authors:** Nasser Shubayr, Meaad Elbashir, Yazeed Alashban, Sarra Ali, Marwan Jafaari, Ali Hendi, Naif Majrashi, Ali Alyami, Nada Alumairi

**Affiliations:** 1 Department of Diagnostic Radiology, Jazan University, Jazan, SAU; 2 Department of Epidemiology and Public Health, Jazan University, Jazan, SAU; 3 Department of Diagnostic Radiography Technology, College of Applied Medical Sciences, Jazan University, Jazan, SAU; 4 Department of Radiological Sciences, College of Applied Medical Sciences, King Saud University, Riyadh, SAU; 5 Department of Radiology, Prince Mohammed bin Nasser Hospital, Jazan, SAU; 6 Department of Medicine, Jazan University, Jazan, SAU

**Keywords:** postoperative, cholecystectomy, weight loss, gallstone, bariatric surgery

## Abstract

Background

Bariatric surgery is performed for accomplishing weight loss, which can save patients from diseases associated with morbid obesity. However, rapid weight loss is one of the most important risk factors contributing to the formation of gallbladder stones. The aim of this study is to investigate the prevalence of gallstone formation among patients in the southern region of Saudi Arabia who underwent bariatric surgery and to evaluate the association between several parameters and gallstone development in these patients.

Methods

A retrospective study was conducted including 57 patients who did not have gallstones in the preoperative abdominal ultrasound examinations. Demographic data, such as age and gender, were obtained along with other parameters like weight loss after surgery, and time elapsed between the surgery to post-surgery US examination. The findings of the US examinations were collected and analyzed. Data were analyzed to obtain descriptive and inferential statistics. A correlation matrix to investigate the dependence between variables was conducted.

Results

Patients in this study underwent either sleeve gastrectomy (87.7% [n = 50]) or gastric banding procedures (12.3% [n = 7]). The occurrence of cholecystectomy was 46% after sleeve gastrectomy and 71.1% after gastric band procedures. The majority of the patients (57.9%) lost weight after surgery in the range of 20 to 40 kg. The time elapses between the surgery to post-surgery ultrasonography examination varied among patients, where less than one year, one to three years, and three to six years accounted for 47.4%, 43.9%, and 8.8%, respectively. Gallstone formation after the surgery was found in 35 (61.4%) of the total cases. Among patients who developed gallbladder stones after bariatric surgery, 62.9%, 28.4%, and 8.6% were within less than one year, one to three years, and three to six years, respectively. The results suggest a statistically significant correlation betweengallstone formation and​​​ the time elapsed after the surgery (P = 0.008) and the type of bariatric surgery (P = 0.006).

Conclusion

The current study found that the overall incidence rate of gallbladder stones after bariatric surgery is 61.4%. The study assumed a possible higher incidence of gallbladder stones following bariatric surgery among the population in the southern region of Saudi Arabia compared to incidence rates reported in the literature. The type of bariatric surgery and the time elapsed after the surgery were found to be of value in predicting the formation of gallstones.

## Introduction

Obesity has been increasing worldwide in both developing and developed countries and affects both children and adults. According to the World Health Organization, Arab Gulf countries have the highest rate of obesity [1]. For instance, Saudi Arabia has been reported in the list of the top 10 countries worldwide, with obesity prevalence among males and females are 31% and 42%, respectively [1]. Rapid urbanization and improvement of living conditions have been reported as the main factors contributing to the prevalence of obesity [2].

Bariatric surgery appears as a possible solution for body weight reduction when patients are unable to sustain weight loss by non-surgical means, and save the patient from the complications and diseases related to morbid obesity [3]. This surgical procedure can be classified according to the type of technique used. So far, there are different procedures for bariatric surgeries including laparoscopic Roux-en-Y gastric bypass, laparoscopic sleeve gastrostomy, laparoscopic adjustable gastric banding, and laparoscopic gastric balloon [4]. Despite the multiple advantages of bariatric surgeries, a review study reported complications such as stenosis, bleeding, portal thrombosis, gallstones, cholecystitis, and leak. Staple line leak at the esophagogastric junction is the most recognized and feared complication, and its prevention remains difficult [5].

Rapid weight loss is one of the most important risk factors contributing to the formation of gallbladder stones [6]. The bile inside the gallbladder stops working to melt fats inside the body as a result of the patient ceasing to eat foods that contain fat, the bile is deposited inside the gallbladder and then turns into gallstones because the gallbladder does not excrete the bile [1].

However, despite the high risk of gallbladder complications after bariatric surgery, there is no commonly accepted therapeutic solution to this problem [7]. There is a broad consensus worldwide that bariatric patients should be screened routinely by ultrasonography (US) for the complications of gallbladder and pathology before undergoing weight loss surgery, and that cholecystectomy should be performed in patients with pathological findings [8]. A study conducted in Saudi Arabia showed that symptomatic cholelithiasis can present soon after sleeve gastrostomy surgery and may require surgical intervention [9]. A number of healthy patients who did not have any history of gallbladder diseases developed gallstones with an acute presentation in some cases after surgery [10]. Cholelithiasis is more common after bariatric surgery, with a high incidence within the first 12-14 months following the procedure [11].

To our knowledge, no studies have investigated the incidence of gallstone formation after bariatric surgery in the southern region of Saudi Arabia. Therefore, the aim of this study is to investigate the prevalence of gallstone formation among patients who underwent bariatric surgery and to evaluate the association between several parameters and gallstone development in these patients.

## Materials and methods

Study area and sample collection

This retrospective descriptive study was conducted in Jazan, the southern region of Saudi Arabia. The study included patients who did not have gallstones in the preoperative abdominal US and those who underwent an annual control US. Patients who had undergone cholecystectomy prior to the bariatric procedures, those who had gallstones in the preoperative US and underwent simultaneous cholecystectomy, and those who did not perform an annual control in the US were excluded. Power analysis was conducted using the G*Power software. Effect size (0.3), alpha error (0.05), Beta error (5%), and degree of freedom (Df=5) were used to calculate the power of the study. The post hoc power was 0.873.

Demographic data such as age and gender were obtained along with other variables like height and weight before and after surgery, and the time elapsed between the surgery to post-surgery US examination. The findings of the US examinations were collected and analyzed.

US procedure

All patients underwent US examination using Philips iU22 US scanners (Philips, Seattle, WA, USA). Patients were informed about all the details and reasons for the examination, and they were advised to fast 4-8 h before the procedure. The postoperative period of all patients was not less than one month. At the time of the procedure, the patient was asked to lie on his back in the supine position and to remove his clothing from the abdominal area to start imaging. The gel was applied to the abdomen around the gallbladder area to help the transducer make secure contact with the body and eliminate air pockets between the transducer and the skin, which can block the sound waves from passing into the body. Using a curvilinear transducer (abdominal probe) with low frequency (2.5 to 5.0 MHz), ideally a large footprint probe, the gallbladder was scanned slowly subcostal and intercostal in the long axis and transverse axis from the funds to the neck, leading to the cystic duct, and then re-scanned in left lateral decubitus or erect positions to ensure the presence of stones.

Statistical analysis

Analysis of the data was conducted using the Statistical Package for the Social Sciences (version 20, IBM Inc., Chicago, IL). Descriptive analysis was performed. A chi-square in the investigation of qualitative data. Fisher’s exact and correlation tests were used when the requirements for chi-square were not met. The results were investigated and presented with confidence intervals of 95%. An α-value of 0.05 was used to determine statistical significance.

## Results

The study included 57 patients who underwent bariatric procedures. As shown in Table 1, female patients account for 87.7% and male patients represent 12.3%. About half of the patients included in this study were in the age group 18 to 30 years, 36.8% in the age group 30 to 40 years, and only 12.3% in the age group 40 to 50 years.

**Table 1:** Socio-demographic distribution of the patients.

Variables	Category	Individuals (%)
Gender	Male	7 (12.3)
	Female	50 (87.7)
Age group (years)	18-30	29 (50.9)
	30-40	21 (36.8)
	40-50	7 (12.3)

As shown in Table 2, all the patients in this study performed either sleeve gastrectomy (87.7% [n = 50]) or gastric banding procedures (12.3% [n = 7]). The occurrence of cholecystectomy was 46% after sleeve gastrectomy and 71.1% after gastric band procedures. The majority of the patients (57.9%) lost weight after surgery in the range of 20 to 40 kg. The time elapses between the surgery to post-surgery US examination varied among patients, where less than one year, one to three years, and three to six years accounted for 47.4%, 43.9%, and 8.8%, respectively. Gallstone formation after the surgery was found in 35 (61.4%) of the total cases. Among patients who developed gallbladder stones after bariatric surgery, 62.9%, 28.4%, and 8.6% were within less than one year, one to three years, and three to six years, respectively.

**Table 2:** Clinical variables related to patients who underwent bariatric surgery.

Variables	Category	Individuals (%)
Type of bariatric surgery	Sleeve gastrectomy	50 (87.7%)
	Gastric banding	7 (12.3%)
Weight loss after surgery (Kg)	10- 20	2 (3.5%)
	20-40	33 (57.9)
	40-80	22 (38.6)
Time elapsed after the surgery (years)	<1	27 (47.4)
	1-3	25 (43.9)
	3-6	5 (8.8)
US detection of the gallstone	Yes	35 (61.4)
	No	22 (38.6)

The association between gallstone formation and several variables including gender, age, amount of weight lost, type of bariatric surgery, and time elapsed after the surgery, is illustrated in Table 3. The results suggest a statistically significant correlation between gallstone formation with the time elapsed after the surgery and the type of bariatric surgery. Also, there was a statistically significant correlation between the amount of weight loss and gender.

**Table 3:** A correlation matrix between gallbladder formation, gender, age, amount of weight lost, type of bariatric surgery, and times elapsed after surgery. * p < 0.05, ** p < 0.01, *** p < 0.001

	Gallstone formation	Age	Gender	Weight lost	Time elapsed after the surgery	Type of bariatric surgery
Gallstone formation	—					
Age	0.43	—				
Gender	0.164	0.681	—			
Weight lost	0.919	0.97	0.003**	—		
Time elapsed after the surgery	0.008**	0.798	0.154	0.698	—	
Type of bariatric surgery	0.006**	0.28	0.299	0.091	0.154	—

## Discussion

Obesity prevalence is commonly reported worldwide and Saudi Arabia is among the top ten worldwide [1]. Bariatric surgeries can be a possible solution for rapid weight loss, but also can contribute to the formation of gallbladder stones [3]. This study investigated the prevalence of gallstone formation among patients who underwent bariatric surgery in the southern region of Saudi Arabia.

Multiple studies have revealed a higher rate of gallstone formation and biliary sludge in patients performing bariatric procedures compared to the normal population, with incidence rates ranging between 10% and 28% for gallstones or biliary sludge [12,13]. The current study found that the incidence rate of gallbladder stones post-bariatric surgery among the Saudi population is 61.4%. These results are higher than the incidence rates reported in the literature. A study in North America indicated an overall 29.8% gallbladder stones formation post-bariatric surgery [14]. Another study in Turkey reported an incidence rate of both symptomatic and asymptomatic gallstones to be 20.7% one-year post-bariatric surgery [15]. With Saudis having a higher body fat proportion compared to Caucasians and potentially different risks of developing gallstones [16,17], the current study assumed a possible higher incidence of gallstone disease following bariatric surgery among the Saudi population. Identifying predictive variables for the formation of gallstones post-bariatric procedures is significant for the selection of patients for specific prophylactic interventions [18].

Fast weight loss increases gallstone formation due to the increase of both cholesterol saturations in bile and biliary mucin concentration in the gallbladder [19,20]. The current study shows no statistically significant correlation between the amount of weight loss post-bariatric procedures and gallstone formation. These findings are not consistent with several previous studies [21,22]. However, several other studies found the amount of weight loss post-bariatric procedures to be not related to the formation of gallstones [15,23].

The present study found that the majority of patients who developed gallbladder stones after bariatric surgery were within less than one-year post-surgery. There was a statistically significant correlation between gallstone formation and time elapsed after the surgery. These findings are consistent with other studies in the literature where the majority of gallbladder stones appeared within 12 months post-surgery [12,24]. Studies on bariatric surgery procedures estimate one-year gallstone development rates between 30% and 53% [19,20,25-28].

Patients in this study underwent either sleeve gastrectomy or gastric banding procedures. There was a statistically significant correlation between gallstone formation and the type of bariatric surgery. The occurrence of cholecystectomy was 46% after sleeve gastrectomy and 71.1% after gastric band procedures. It has been proposed that the chance of developing gallstones is directly related to the surgical procedure used for the treatment of obesity. Different types of bariatric surgeries further increased the risk by changing the enterohepatic circulation and the ordinary physiology of the gallbladder [25]. Restrictive bariatric surgery procedures such as sleeve gastrectomy and gastric banding usually have a relatively lower gallbladder stones formation risk and therefore cholecystectomy compared to other procedures, as gallbladder contraction processes and enterohepatic circulation are not straight affected [12]. A previous study reported that the frequency of cholecystectomy was 10.6% after Roux-en-Y gastric bypass, considerably higher than 2.9% after laparoscopic adjustable gastric band, and 3.5% after laparoscopic sleeve gastrectomy [29].

The limitations of the study include its single-region focus, the limited number of patients, and not testing other factors such as smoking, and alcohol drinking, in addition to whether or not patients have diabetes mellitus, hypertension, or cardiovascular diseases.

## Conclusions

Among the 57 patients, the incidence rate of gallbladder stones post-bariatric surgery was 61.4%. These results are higher than the incidence rates reported in the literature. The majority of patients developed gallbladder stones after bariatric surgery (62.9%) within less than one-year post-surgery. The study assumed a possible higher incidence of gallstone disease following bariatric surgery among the population in the southern region of Saudi Arabia. The type of bariatric surgery and time elapsed after the surgery were found to be of value in predicting the formation of gallstones.
